# Molecular Interactions in Solid Dispersions of Poorly Water-Soluble Drugs

**DOI:** 10.3390/pharmaceutics12080745

**Published:** 2020-08-07

**Authors:** Thao T. D. Tran, Phuong H. L. Tran

**Affiliations:** 1 Institute of Research and Development, Duy Tan University, Danang 550000, Vietnam; trantdinhthao@duytan.edu.vn; 2 The Faculty of Pharmacy, Duy Tan University, Danang 550000, Vietnam; 3 Deakin University, School of Medicine, IMPACT, Institute for Innovation in Physical and Mental Health and Clinical Translation, Geelong, Australia

**Keywords:** solid dispersion, molecular interaction, poorly water-soluble drug, physicochemical characterization, bonding formation, bonding force

## Abstract

Physicochemical characterization is a crucial step for the successful development of solid dispersions, including the determination of drug crystallinity and molecular interactions. Typically, the detection of molecular interactions will assist in the explanation of different drug performances (e.g., dissolution, solubility, stability) in solid dispersions. Various prominent reviews on solid dispersions have been reported recently. However, there is still no overview of recent techniques for evaluating the molecular interactions that occur within solid dispersions of poorly water-soluble drugs. In this review, we aim to overview common methods that have been used for solid dispersions to identify different bond formations and forces via the determination of interaction energy. In addition, a brief background on the important role of molecular interactions will also be described. The summary and discussion of methods used in the determination of molecular interactions will contribute to further developments in solid dispersions, especially for quick and potent drug delivery applications.

## 1. Introduction

Poorly soluble or insoluble drugs result in low absorption, which certainly affects drug bioavailability, especially in oral drug delivery [1,2,3,4,5]. Therefore, many formulation strategies have been developed to overcome the limitations of these drugs [6,7,8,9,10]. Both traditional approaches (e.g., polymorphs, prodrugs, salt formation, and solid dispersions (SDs)) and current nanotechnologies (e.g., solid lipid nanoparticles, nanoprecipitations, and nanoemulsions) have contributed useful techniques and strategies for the development of formulations of poorly water-soluble drugs [8,11,12,13,14,15,16,17,18,19,20,21,22,23]. Among them, the development of SDs (even in combination with nanotechnology) is still being heavily investigated to improve the drug solubility, dissolution rate and stability of poorly water-soluble drugs [24,25,26,27,28].

**Figure 1:**
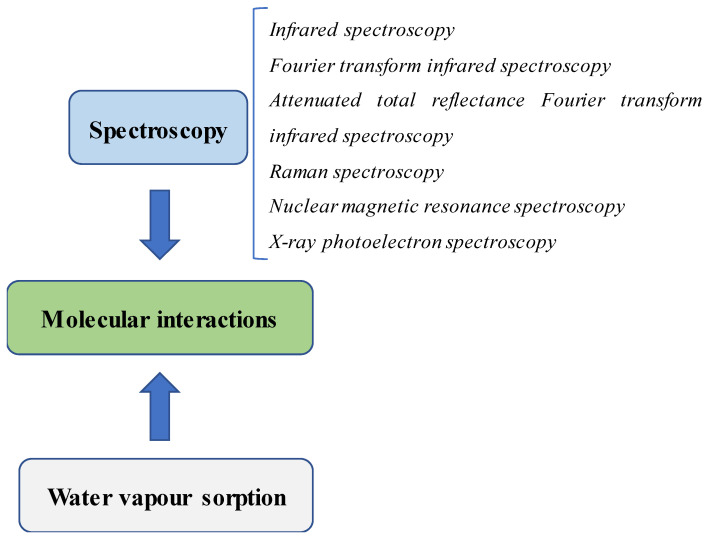
Common techniques used in the detection of molecular interactions in SDs.

Physicochemical analysis is necessary to characterize SDs during their preparation. Many pieces of information can be obtained, such as how the polymer affects drug crystallinity, how the molecular interactions between the drug and the components in the formulation occur and how the binding force is generated. The data from these studies will be useful in the explanation of different drug performances and will contribute to the selection of the best formulation in drug development. Therefore, the scrutinization of the molecular interactions in SDs is critical information that needs extensive investigation. This review will provide insights into common methods for the detection of the molecular interactions in SDs used in recent studies. Moreover, the strategies used in the determination of binding forces will also be discussed as they are helpful in differentiating the different binding interactions between formulations. Figure 1 illustrates common techniques used in the detection of molecular interactions in SDs.

## 2. How Molecular Interactions Are Important in SDs

In SD systems, typically a poorly water-soluble drug is dispersed in a carrier or a mixture of carrier and additives [29,30]. Details of several generations of SDs were well described in previous reviews [31,32]. While in the first generation of SD carriers are commonly in crystalline state, amorphous polymers are utilized in the second generation of SD to transform a drug crystal to an amorphous form [31,32]. For the third and fourth generations of SDs, researchers take into account adding additives (e.g., surfactants, pH-modifiers) in formulations for further improving drug bioavailability. Particularly, insoluble carriers or swellable polymers have been suggested in the fourth generation of SDs for a constant drug release rate [31,32,33]. 

**Figure 2:**
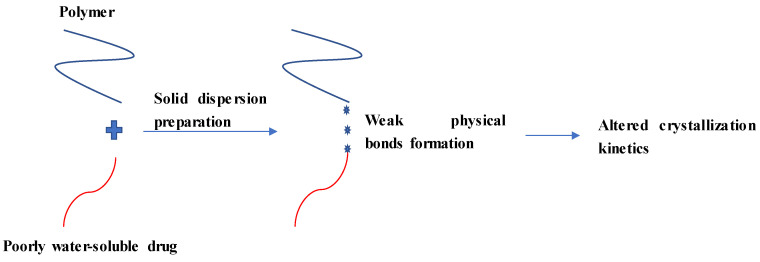
Illustrations of typical molecular interactions in solid dispersions and their effects on drug crystallinity.

Once a poorly water-soluble drug is dispersed in a polymer for SD formation, a weak physical bond may be formed between these components to modulate drug release (Figure 2). Weak physical bonds (formed by non-covalent interactions) such as hydrogen bonds, ionic bonds, van der Waals, dipole-dipole interactions and acid-base interactions are common interactions occurred between components in SDs [26,34,35,36,37,38]. Among them, hydrogen bonding formation is typically observed in SDs [39,40,41,42]. The formation of these bonds between a drug and one of the SD components may prevent self-association between drug molecules, leading to changes in the crystallization kinetics [43].

The interaction at the molecular level between a drug and a polymer is crucial to explain the mechanism of drug release and stability in SD systems. Generally, these interactions may maintain a drug in an amorphous form during the dissolution process as well as during storage [44,45]. In particular, hydrogen bonding has been demonstrated to be an important factor in improving amorphous stability [46,47].

The molecular interaction (for example, between griseofulvin and hydroxypropyl methylcellulose acetate succinate) is even maintained in the liquid state, as demonstrated in the study of Al-Obaidi et al. [44]. In summary, the interaction between a drug and a polymer affects the solubility, dissolution and physical stability of a drug in its SD. Although different types of molecular interactions may occur within SDs, a selection of common methods to clarify the interactions, which will be described below, depends on the physicochemical properties of SDs rather than their generations.

## 3. Spectroscopic Techniques Used in the Investigation of Molecular Interactions

### 3.1. Infrared Spectroscopy (IR)

**Figure 3:**
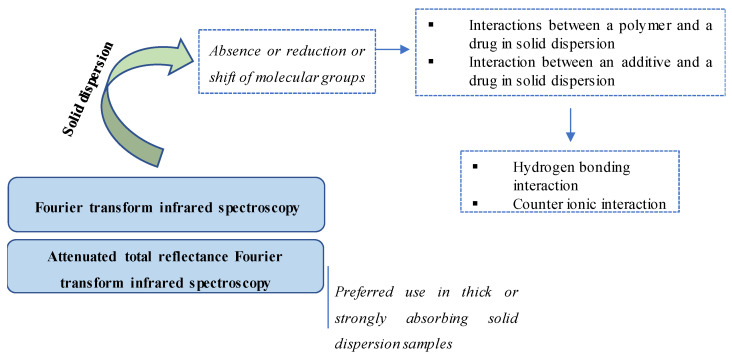
Illustration of IR methods and their detection in molecular interactions in SDs.

IR has been widely used as one of the most effective techniques to identify a chemical or detect impurities because it can obtain the structural information of a wide range of compounds [48,49,50,51]. Figure 3 illustrates different IR methods and their detection of molecular interactions in SDs. In SD studies, IR is a common technique for the determination of molecular interactions between components, particularly those between a drug and an SD component [17,26,52,53,54,55,56]. Indeed, the use of IR was recommended in early studies on SD to identify the interaction between a model drug and a polymer. For example, Tachibana et al. used IR in 1965 to evaluate the molecular dispersion of β-carotene in polyvinyl pyrrolidone [57]. 

The most common type of IR is Fourier transform infrared (FTIR) spectroscopy, which transforms the recorded data into a spectrum [58,59]. In principle, FTIR is used to investigate and compare the spectra of individual components in mixtures of samples (including SD samples) [60,61]. The absence, reduction or shift in the spectra of molecular groups would suggest the occurrence of molecular interactions in SDs [57,62,63].

For example, in a study of SDs containing esomeprazole, the hydrogen bonding interactions were determined by FTIR [34]. Specifically, the binary SD of esomeprazole and hydroxypropyl methylcellulose (HPMC) showed a shift in the spectra of the sulfonyl group (S=O) and amino group (C=N), which was demonstrated to be the result of hydrogen bonding interactions [34]. However, ternary SDs with added pH modifiers, showed strong hydrogen bonding, which was demonstrated by the reduction of the stretching vibration frequency of those peaks [34,64]. Similarly, in a study of SDs containing zein and isradipine, the disappearance of C=O and NH groups in the FTIR spectrum indicated hydrogen bond formation between the model drug and the polymer in the SD [65].

The peak height ratio can be used to quantify the level of hydrogen bonding. Indeed, Ozeki et al. utilized this information in the evaluation of an increase in the ratio of hydrogen bonding between flurbiprofen and poly(ethylene oxide) [66]. Specifically, while the carbonyl stretching band of flurbiprofen was shown at 1703 cm^−1^ in the FTIR spectrum, the new peak at 1736 cm^−1^ confirmed hydrogen bonding formation [66]. The authors showed that the increase in the peak height ratio was correlated with the release rate of flurbiprofen and the increase in the ratio of hydrogen bonding [66].

FTIR can also detect counterionic interactions. In a study of SDs using ionic and non-ionic polymers, Sarode et al. found that the carbonyl groups (with peaks between 1670 cm^−1^ and 1800 cm^−1^) of the drugs (indomethacin and itraconazole) were stretched when the drugs were included in SD formulations [35]. In particular, significant stretching was observed with SDs using ionic polymers [35]. Therefore, counterionic interactions occurred in SDs, particularly a stronger interaction between the weakly acidic drug and the cationic polymer [35].

In the case of thick or strongly absorbing SD samples, attenuated total reflectance FTIR (ATR-FTIR) spectroscopy, which has been utilized widely as a powerful technique in recent SD studies and the analysis of biopharmaceuticals, is recommended to overcome the intense peaks that are produced by these samples [67,68,69,70]. This system consists of an FTIR spectroscope and an ATR accessory to create an evanescent wave that is then directed into the sample [71]. Contact between an ATR crystal surface and a sample will affect the quality of measurement [72]. The investigation of any shifted peaks or changes in intensity is similar to FTIR. For instance, in a study of intermolecular interactions in SDs using copovidone carriers, ATR-FTIR was used to examine the hydrogen bonding between the mixture of polymers ((poly(vinylpyrrolidone-vinyl acetate) copolymer and Plasdone S-630)) and indomethacin [73]. Indeed, a shift in the amide carbonyl peak (1672 cm^−1^ to 1680 cm^−1^) demonstrated a hydrogen bond formation in SDs [73].

In addition to the detection of the interaction with polymers, ATR-FTIR can also detect the interactions between a model drug and an additive in an SD [74]. In a study of ternary SDs containing naftopidil/fumaric acid/d-α-tocopherol polyethylene glycol 1000 succinate (TPGS), the interaction between naftopidil and fumaric acid was discovered by comparing binary SDs and ternary SDs [74]. Specifically, C=O stretching (1737.3 cm^−1^) did not change in the binary SDs, while C=O stretching was observed at 1737.3/1697.7 cm^−1^, which was attributed to the C=O peaks of TPGS and fumaric acid, respectively [74]. In short, the shift in the C=O peak of fumaric acid in SDs demonstrated hydrogen bond formation between naftopidil and fumaric acid [74].

### 3.2. Raman Spectroscopy

It should be noted that the presence of functional groups in polymers (e.g., carbonyl groups), which are similar to those in a model drug, could interfere with methods to distinguish the spectra of a drug and a polymer [73]. Therefore, combination methods are often used to identify molecular interactions [73]. Raman spectroscopy is an example of these techniques. Indeed, the use of Raman spectroscopy can distinguish differences in short-range ordering [75]. Moreover, aqueous samples can be studied with Raman spectroscopy [75,76]. Table 1 summarizes key characters complementary to each other of Raman spectroscopy, FTIR and ATR-FTIR.

One of the remarkable advantages of Raman spectroscopy is the estimation of drug crystallinity in SDs [77]. Generally, a drug crystal is represented by the defined peaks in the Raman spectrum because of the phonon region pattern of the crystalline form [78,79,80,81]. In contrast, a broad spectrum is observed when the drug is in an amorphous state [78,79,80,81]. In 1998, Taylor et al. used Raman spectroscopy to quantitate the degree of indomethacin crystallinity in mixtures of its crystal and amorphous states [82]. In detail, the authors utilized the peak intensity ratio of the crystal and amorphous spectra to establish a correlation curve [82]. In a later study, Okumura et al. developed a calibration model using chemometric FT-Raman spectroscopy that could evaluate the small differences in microcrystallinity in an indomethacin tablet (both on the surface and in cross-section) [83].

The determination of drug crystallinity could also be made during the preparation of an SD, which was shown in the study of Saerens et al. [84]. In this study, the hot-melt extrusion process was used to produce SDs [84]. During this process, a Raman spectrometer probe, which was built into the extrusion die, monitored the samples before they were forced through the die [84]. Similar to IR spectroscopy, the peak shifts in the spectra of the SDs indicate the interaction between a drug and a polymer [84]. The existence hydrogen bonding forces, formed between the drug and a polymer, are typically inferred from the blueshift and redshift in Raman spectra [85,86].

### 3.3. Nuclear Magnetic Resonance (NMR) Spectroscopy

NMR is another powerful technique that can be used to perform high sensitivity detection and obtain quantitative information at the molecular level [87]. In general, NMR monitors possible changes of electron density around specific interacting atoms to detect molecular interactions at the atomic level [88,89]. For instance, the observation of chemical shifts in ^1^H NMR spectra can reveal hydrogen bond formation in SDs [90,91,92,93]. Typically, the chemical shift is attributed to the change in electron density around the proton caused by hydrogen bond formation [91,94]. Potential disadvantages of NMR involve the requirement of a high-field NMR spectrometer with a combination of multi-channel probe and multiple magnetic fields in certain circumstances [95].

The level of hydrogen bonding can also be detected by NMR spectroscopy. For example, in the study of Karavas et al., the authors used ^1^H-NMR to compare the interaction between felodipine-polyethylene glycol and felodipine-polyvinyl pyrrolidone in SDs [96]. Although FTIR could indicate the hydrogen bonds between felodipine and the two polymers, ^1^H-NMR was used to evaluate the intensity of the felodipine-polymer interactions [96]. Specifically, they observed the secondary amino group of felodipine at 5.75 ppm [96]. This peak was shifted to 6.39 ppm, 6.57 ppm and 6.62 ppm when SDs with polyvinyl pyrrolidone were manufactured with 10%, 20% and 50% felodipine, respectively [96]. In contrast, this peak was almost stable as the amount of felodipine in the SDs increased further [96]. Therefore, ^1^H-NMR could indicate a stronger hydrogen bonding interaction when using polyvinyl pyrrolidone in SDs than when using polyethylene glycol [96].

Solid-state NMR (ssNMR) spectroscopy can also detect hydrogen bond formation in SDs. In a study of SDs containing nifedipine, ^13^C ssNMR was used to observe the difference in the C=O peaks of nifedipine in SDs [97]. While the C=O peaks in SDs with Eudragit^®^ were observed at 170 ppm, the SDs with HPMC showed the C=O peak at 168 ppm, which indicated a hydrogen bonding formation as a lower magnetic field was observed compared to the non-hydrogen bond [97,98,99].

### 3.4. X-ray Photoelectron Spectroscopy (XPS)

In terms of characterizing polymer surfaces, XPS is more sensitive than NMR and FTIR [100]. XPS can provide valuable information such as layer structure, elemental distribution, and chemical bonding within material surfaces (even in nanostructure materials) [100,101]. In the study of Maniruzzaman et al., the authors found that although molecular modelling was able to predict two types of hydrogen bonds, XPS could indicate the exact interaction between a cationic drug and an anionic polymer via intermolecular ionic interactions to form a hydrogen bond [100].

In particular, XPS is considered a powerful technique in the evaluation of acid-base interactions because it can detect the binding energy shifts of the selected atoms resulting from protonation [102,103]. For instance, Song et al. utilized XPS to identify the interactions in SDs containing polystyrene sulfonic acid/apatinib or polystyrene sulfonic acid/gefitinib [36]. By detecting the increase in binding energy of the basic nitrogen atoms of the drugs, XPS provided information about the protonation of these nitrogen atoms [36]. This study also noted that XPS is unable to distinguish different NH groups (e.g., aliphatic secondary amine NH vs. aniline NH), although XPS can detect local protonation [36]. Therefore, a combination of XPS and NMR spectroscopy was suggested for detecting acid-base interactions in SDs [36]. However, XPS alone has been used to detect acid-base interactions. For example, in another study by the same research group above, the tertiary amine of lumefantrine was used to evaluate the extent of protonation through interactions with acidic polymers (hydroxypropyl methylcellulose phthalate, hydroxypropyl methylcellulose acetate succinate, poly(methacrylic acid-co-ethyl acrylate), polystyrene sulfonic acid and polyacrylic acid) using XPS [102].

## 4. Water Vapour Sorption (WPS)

WPS is a method used to examine the water sorption behaviour of a powder to investigate its affinity towards water [104]. By measuring the deviation in water sorption, interactions in the mixture arising from the masking of monolayer water-binding sites can be deduced [105]. In other words, the interactions in SDs affect the water sorption of polymers and active pharmaceutical ingredients. For example, Costantino et al. utilized gravimetric sorption analysis to calculate the water monolayer in lyophilized protein-sugar systems [105]. Specifically, the water monolayer was lower in the entire system than in component in the system (protein and sugar) [105]. These data indicated that the interaction between sugar and protein occurred in the solid-state, leading to a decrease in the availability of water-binding sites [105]. In a study of SDs containing hydrophobic drugs (indomethacin, ursodeoxycholic acid or indapamide) and poly(vinylpyrrolidone), a similar result was observed with water vapour sorption [106].

However, it should be noted that the hydrogen bonding interactions in SDs are not sufficient to affect the water sorption of individual components [107]. Zhang et al. demonstrated that two amorphous SD systems (sucrose-poly(vinylpyrrolidone) and trehalose-poly(vinylpyrrolidone)) showed a similar water vapour sorption as the pure components, although there were hydrogen bonding interactions present in the SDs [107]. Because of this limitation, water vapour sorption only has predictive potential [104].

## 5. Conclusions

In the preparation of SDs, it is important to choose a method to evaluate the interactions between a drug and a polymer (or other components). Spectroscopic methods including IR, Raman, NMR and XPS are the most common methods used to identify the intermolecular interactions in SDs. Although these methods can indicate the types of bonding formations or even quantify the interactions themselves, the methods used in the determination of interaction energy such as molecular modelling and quantum chemical calculation would be useful tools in determining the level of bonding formation as well as the prediction of bonding type [108,109]. With regard to future perspectives in the characterization of SDs, the development of advanced analytical equipment/methods that can distinguish and quantify molecular interactions will play a key role in the development of SDs.

## Figures and Tables

**Table 1:** Key characters complementary to each other of Raman spectroscopy, FTIR (Fourier transform infrared spectroscopy) and ATR-FTIR (attenuated total reflectance FTIR).

Methods	Key Characteristics	References
FTIR	Application for wide range of compounds Quantification of hydrogen bonding level Detection of counterionic interactions	[35,48,49,50,66]
ATR-FTIR	Preferred use in recent studies on SDs (solid dispersions), especially for thick or strongly absorbing solid dispersion samples.	[67,68,69,70]
Raman spectroscopy	Distinguishing differences in short-range ordering Can be studies with aqueous samples Estimation of drug crystallinity in SDs, possible during the preparation of an SD	[75,76]
